# High Levels of Genetic Variation in MHC-Linked Microsatellite Markers from Native Chicken Breeds

**DOI:** 10.3390/genes12020240

**Published:** 2021-02-08

**Authors:** Prabuddha Manjula, Minjun Kim, Sunghyun Cho, Dongwon Seo, Jun Heon Lee

**Affiliations:** Division of Animal and Dairy Science, Chungnam National University, Daejeon 34134, Korea; prabuddhamanjula@yahoo.com (P.M.); mjkim6023@o.cnu.ac.kr (M.K.); cshcshh@cnu.ac.kr (S.C.); seotuna@gmail.com (D.S.)

**Keywords:** MHC-B, haplotypes, microsatellites, heterozygosity, production system

## Abstract

The major histocompatibility complex (MHC) is a highly polymorphic gene region that regulates cellular communication in all specific immune responses. In this study, we investigated 11 microsatellite (MS) markers in the MHC-*B* region of chicken populations from four countries: Sri Lanka, Bangladesh, South Korea, and Nigeria. The MS markers were divided into two sets. Set 1 included five novel MS markers, which we assessed using 192 samples from 21 populations. Set 2 included six previously reported markers, which we assessed using 881 samples from 29 populations. The Set 1 MS markers had lower polymorphism (polymorphic information content (PIC) < 0.5) than the Set 2 markers (PIC = 0.4–0.9). In all populations, the LEI0258 marker was the most polymorphic, with a total of 38 alleles (PIC = 0.912, expected heterozygosity (H_e_) = 0.918). Local populations from Sri Lanka, Bangladesh, and Nigeria had higher allele diversity and more haplotypes for Set 2 MS markers than Korean and commercial populations. The Sri Lankan Karuwalagaswewa village population had the highest MHC diversity (mean allele number = 8.17, H_e_ = 0.657), whereas the white leghorn population had the lowest (mean allele number = 2.33, H_e_ = 0.342). A total of 409 haplotypes (89 shared and 320 unique), with a range of 4 (Rhode Island red) to 46 (Karuwalagaswewa village (TA)), were identified. Among the shared haplotypes, the B21-like haplotype was identified in 15 populations. The genetic relationship observed in a neighbour-joining tree based on the D_A_ distance agreed with the breeding histories and geographic separations. The results indicated high MHC diversity in the local chicken populations. The difference in the allelic pattern among populations presumably reflects the effects of different genotypes, environments, geographic variation, and breeding policies in each country. The selection of MHC allele in domestic poultry can vary due to intensification of poultry production. Preserved MHC diversity in local chicken provides a great opportunity for future studies that address the relationships between MHC polymorphisms and differential immune responses.

## 1. Introduction

Characterising the non-neutral genomic region that is likely to be under natural selection can reflect evolutionarily relevant and adaptive processes within and between populations [1]. To date, many studies have managed to quantify adaptively important gene families. They have found that diversity is maintained by forces of natural selection, such as balancing selection or positive selection. The major histocompatibility complex (MHC) genes in vertebrates are among the gene families that play a critical role in host immunity [2,3,4,5,6,7,8].

In chicken, the MHC is located on the q-arm of micro-chromosome 16 (GGA16). Compared to the MHC of higher vertebrates, such as humans, swine, and mice, the chicken MHC gene arrangement is unique. Classic MHC genes are densely arranged in the MHC-*B* region, which is separated from the non-classic MHC-*B*-like region called the MHC-*Y*. The intergenic and intron regions of these genes are shorter than any other part of the genome; therefore, genes have a high linkage disequilibrium. Because of this specific gene arrangement, the chicken MHC is also known as a minimal essential region [2,3].

MHC-*B* molecules are associated with resistance or susceptibility to highly pathogenic viruses and bacterial diseases in chicken [9,10,11]. Identifying the MHC-*B* haplotypes and their diversity in different chicken breeds under different environmental conditions and production systems is useful for assessing the diversity of these breeds in relation to their immune responses to various diseases under natural and artificial influences.

Native chicken breeds in tropical countries have a strong capacity to survive under harsh environmental conditions and also have high resistance to disease [12,13]. In local production systems, village chickens are kept in challenging environments and are constantly exposed to numerous viruses, bacteria, and parasite-induced diseases. Because host genetic diversity plays an important role in buffering individuals or populations against pathogens and widespread epidemics, their genes confer resistance to a number of major poultry diseases, such as Marek’s disease, avian influenza, Rous sarcoma tumour virus, and fowl cholera. Unlike MHC-*B* genes, MHC-*Y* polymorphism that showed disease resistance is not well understood. Only a few disease trials have been conducted to see the response of MHC-*Y* polymorphisms on Marek’s disease and have reported varied results with no strong impacts but possible influences [11]. A recent study by Seroussi et al. [14] reported a crucial role of MHC genes in immunity, particularly the avian tumour-necrosis factor (TNF) gene mapped to the MHC-*Y* region in chicken. Selecting birds with genotypes that are functionally associated with sound immune responses has the potential to control the occurrence of disease in chicken production systems, thus improving correlated production traits [9,11,15].

Microsatellite (MS) markers that are closely linked to a gene under selection may be chosen through genetic hitchhiking. MHC-linked MS markers can provide simple and cost-effective genotyping for assessing MHC diversity and identifying haplotypes. Many MS markers have been identified in the human leukocyte antigen (HLA) and swine leukocyte antigen (SLA) complexes, with extensive diversity in the classic class I, II, and III regions [16,17,18,19]. However, to date, only six MS markers have been reported in the chicken MHC-*B* region. Among those markers, the variable number tandem repeat marker LEI0258 has been reported in many MHC diversity studies [19,20,21,22,23,24].

There have been few studies of the other MS markers in the chicken MHC region. Among the reported markers, variations in MHC-T, MCW0312, MHC-D, MCW0371, and MCW0370 [19,20,21,22,23] have been reported in very few chicken populations. The LEI0258 marker is consistently more polymorphic and therefore is more frequently used in diversity analyses and marker–trait association studies. It has been suggested that LEI0258 is an important genetic marker that can be used to predict diversity at MHC loci [22,24,25,26,27,28]. The availability of updated MHC-*B* sequence information has facilitated the identification of other novel MS markers in the MHC-*B* class I and II regions. Further studies of their polymorphism in a wide range of breeds would be useful.

The main objective of this study was to develop an MS marker panel by combining previously reported markers and new MS markers in the MHC-*B* region (the 148.5 kb region of MHC-*B* that encloses the BG8 to C4 genes) to investigate MHC diversity in 29 different native chicken breeds/ecotypes from four countries (Sri Lanka, Bangladesh, Korea, and Nigeria) that manage chickens under different production systems and breeding practices. Furthermore, identification of MHC haplotypes (low resolution) and their distribution in global chicken populations will be required for planned disease association studies in native chicken breeds.

## 2. Materials and Methods

### 2.1. Samples and DNA Extraction

A total of 29 populations, including samples from Sri Lanka, Bangladesh, Nigeria, and South Korea and commercial layer and broiler populations, were used in the study. The breeds/ecotypes had varied histories of origin, breeding strategies, and selection criteria and had been raised under different management systems. We categorised them into three main management systems (extensive/semi-intensive, intensive system for native chicken, and intensive system for commercial chicken) based on the prevailing production systems of the four countries (Supplementary Table S1).

Details of the populations and samples are provided in Supplementary Table S2. Briefly, blood samples from Sri Lankan indigenous chickens were collected from three geographically distinct sites: University of Peradeniya farm, Udaperadeniya, Kandy (KA; Central Province, Sri Lanka); Udapussellawa village (NU; Central Province, Sri Lanka); and Karuwalagaswewa village (TA; Northwestern Province, Sri Lanka). For Bangladeshi chicken, we used the same sample set as used by Rashid et al. [29], which included five different breeds: Aseel chicken (AS), hilly chicken (HI), non-descript common deshi (ND), naked neck (NN), and wild red jungle fowl (JF). Nigerian local chicken samples (NIG) used in this study were collected from Umuobasi village (Osisioma Ngwa area, Abia State, Nigeria). We used 12 South Korean native chicken breeds: the six native lines used by Manjula et al. [30], a Korean native black chicken population called “Ogye” sampled from Yeonsan Ogye Jisan farm in Nonsan city (YOG), four Korean native commercial hybrid lines from the Hanhyup private breeding company (CC1, CC2, CC3, and CC4), and a hybrid population from the National Institute of Animal Science (NIAS; YCC). Two imported and adapted standard breeds, Rhode Island rock (RIR) and white leghorn (WL), were sampled at NIAS. Two commercial layer lines and four commercial broiler lines were collected from commercial private farms in Korea. All Korean native chicken populations were maintained under an intensive production system, except for the YOG population, which is usually maintained under a semi-intensive production system, with natural mating and artificial incubations.

The DNA of seven MHC-*B* standard samples (B2, B5, B12, B13, B15, B19, and B21) of the Avian Disease and Oncology Laboratory (ADOL) chicken line were received from HY-Line International (West Des Moines, IA, USA) and were used as controls for the study. The genomic DNA of Sri Lankan and Bangladeshi chicken samples was extracted from Flinders Technology Associates cards following the standard procedure. For Nigerian chicken, a rapid and accurate protocol was used to isolate the genomic DNA from the fresh feathers kept for 5 days at room temperature using a Prime prep-genomic DNA extraction kit for tissue (GeNet Bio, Daejeon, Korea). The genomic DNA of Korean chickens was obtained from whole blood samples using a Prime prep-genomic DNA extraction kit for whole blood (GeNet Bio, Daejeon, Korea).

### 2.2. Ms Marker Identification and Primer Design

**Figure 1:**
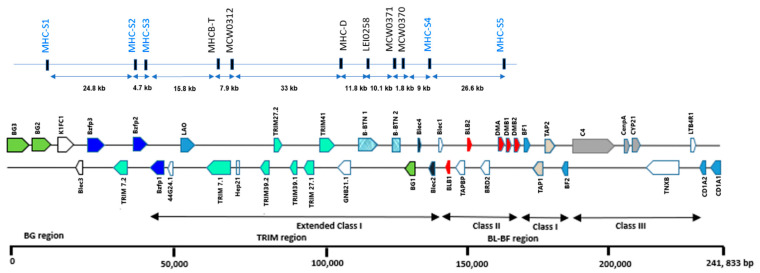
A graphical representation of microsatellite markers identified in chicken major histocompatibility complex (MHC)-*B* region. The loci in blue colour letters are novel microsatellite (MS) markers identified in this study (Set 1) whereas the markers in black colour letters are reported in the literature (Set 2). Physical positions and distance between the markers (not in the exact scale) are based on the sequence of GenBank accession number: NC_006103.5.

To obtain new MS markers and confirm the previously reported MSs, we analysed the ~240 kb region of MHC-*B* using three reference genomic sequences (Gene ID: NT_455973.1, AL023516.3, and AB268588.1) and the chromosome 16 (GGA16) sequence information for the *Gallus gallus* 6.0 assembly available in the National Center for Biotechnology Information (NCBI) database. We used a version of the MS tandem repeat finder available in the tandem repeats database (Tandem.bu.edu/cgi-bin/trdb/trdb.exe) [31] to identify all MSs in the MHC-*B* region, which enclosed the gene region from BG to CD1. The results were filtered to obtain the repeat motifs less than 20 bp in size (mono-, di-, tri-, tetra-, penta-, etc.). Among the MS markers, loci with perfect and imperfect repeat patterns reported within the intron region of MHC-*B* genes were selected. Five new MS markers (Set 1: MHC-S1, MHC-S2, MHC-S3, MHC-S4, and MHC-S5) and six previously reported MS markers (Set 2: MHC-T, MCW0312, MHC-D, MCW0371, MCW370, and LEI0258) were used (Figure 1). All Set 2 markers and four new loci belonged to the extended MHC class I (*TRIM/Blec* gene region). Only one new locus (MHC-S5) belonged to the MHC class II (in the *DMB1* gene; Figure 1). The minimum gap between two markers was 1.8 kb (between MCW0370 and MCW0371), whereas the maximum gap was 148.5 kb (between MHC-S1 and MHC-S5).

Polymerase chain reaction (PCR) primers for new MS loci were designed with the Primer3 program and validated by BLAST against the chicken genome and MHC-*B* sequence (AB268588) available in the NCBI database to ensure high specificity to the target sequence. First, we verified the utility of the normal oligo primers for the 11 MS markers using the MHC-*B* standard (MHC homozygous) samples and selected chicken samples from six breeds. Then, we performed fluorescence-based allele identification, with the 5′ end of each forward primer labelled with standard fluorescence colours (FAM, VIC, HEX, and NED; Supplementary Table S3).

### 2.3. PCR Amplification and Genotyping

The PCR procedure and primers for the Set 2 markers were consistent with the previously performed procedure, with a few modifications [19,22,23]. These six markers were genotyped using 881 samples from 29 populations.

We genotyped the five new loci identified in this study using a set of 192 samples from 21 populations. Initially, we performed a single PCR reaction for each marker and then a multiplex PCR reaction based on its fragment sizes and fluorescence colours. The following optimised PCR procedure was used for all loci. The PCR master mixture consisted of 1 µL of 50 ng/µL genomic DNA; 10 µL HS prime Taq premix (2×; GeNet Bio); 0.5 µL 5 pmol of each forward and reverse primer; and triple distilled water, which was used to adjust the volume to 20 µL. The PCR thermocycler setup included an initial denaturation for 3 min at 94 °C, followed by 34 cycles of denaturation at 94 °C for 0.45 s, annealing at 60–62 °C for 45 s, at 72 °C for 45 s, and a final extension at 72 °C for 60 min. The PCR products for each sample were verified with 3% agarose gel containing ethidium bromide (Supplementary Figure S1). Based on the electrophoresis results, 1 µL PCR product was diluted (1:100, 1:50) with triple distilled water to reduce the pull-up during capillary electrophoresis. A mixture of 1 µL of each diluted PCR product was mixed with 0.1 µL Gene-Scan LIZ 500 size standards and 9.9 µL Hi-Di formamide solution and analysed by capillary electrophoresis with an ABI-3730 DNA sequencer (Applied Biosystems, Foster City, CA, USA) at the Neogene laboratory (Korea).

Raw data were analysed with Genemapper 3.4 (Applied Biosystems), and allele binning was conducted with the power function of Tandem [32]. As a control, we used the allele sizes of seven MHC-*B* standards from the ADOL line [22], and the sequence information of B standard haplotypes from Hosomichi et al. [33] and Shiina et al. [34] was used to validate and adjust the binning size. The LEI0258 alleles were further sequenced to confirm their actual fragment sizes and adjust the allele sizes in the binning process. A total of 21 fragment sizes were selected for PCR amplification and sequencing using a new primer pair as described previously [22,26].

### 2.4. Population Genetics Statistics

Raw data were initially checked for possible errors in allele sizes with an MS tool kit program. A test for deviation from the Hardy–Weinberg equilibrium (HWE) for all loci was conducted with Genepop [35] and GenAlEx [36]. We calculated the following population genetics statistics for the 11 markers for each population using the GenAlEx program in Excel: allele frequency, number of alleles (Na), effective allele size (Ne), private allele size (Npa), observed (H_o_) and expected heterozygosity (H_e_), and heterozygosity excess (fixation index = 1 − [H_o_/H_e_]). We calculated the polymorphic information content (PIC), H_o_, and the null allele frequency of each marker using Cervus [37]. We calculated allele richness (AR) using the hierarchical rarefaction method (with a minimum number of animals, n = 32) available in HP-RARE software for rarefaction of private alleles and hierarchical sampling designs [38].

#### MS Marker Diversity within and between Populations

We analysed differences in allele frequency distribution within and among populations under two scenarios using analysis of molecular variance (AMOVA), using an exact test implemented in Arlequin [39]. Populations were grouped based on their country of origin, and F statistics were used to estimate the proportion of genetic variation within populations (F_ST_), variation among populations within groups (F_SC_), and variation among groups (F_CT_). The significance of these F statistic analogues was evaluated with 1000 random permutations. Allele number, AR, and heterozygosity difference among the groups, which were separated based on their origin and production systems, were compared with independent t tests.

To perform genetic clustering of the individuals based on the MHC-linked MS markers, we calculated the Nei unbiased genetic distance (Nei D) and genetic identity value (Nei I) using GenAlEx.

We created a neighbour-joining (NJ) tree based on the Nei’s D_A_ distance [40] using the POPTREEW Web interface (accessed at http://www.med.kagawau.ac.jp/~genomelb/takezaki/poptreew/index.html (accessed on 15 November 2020)) [41].

### 2.5. Haplotype Construction

The MHC-*B* regions of MS-based haplotypes were typed with the Set 2 markers located in the extended class I. First, the haplotypes of seven standard MHC-*B* samples were constructed manually. Haplotypes were then constructed in each population using homozygous individuals. If an individual’s genotypes were not described by any of the homozygous haplotypes, all possible haplotype pairs for ambiguous data (gametic phase unknown) were identified with the expected maximum likelihood algorithm and Bayesian algorithm methods in Arlequin [39].

## 3. Results

### 3.1. MHC-B MS Marker Polymorphisms

The amplified MHC-linked markers represented two classic classes of chicken MHC-*B*: extended class I and class II (Figure 1). The overall polymorphic characteristics of the 11 MS markers, Set 1 (MHC-S1 to MHC-S5) and Set 2 (MHC-T to MCW0370), are summarised in Table 1. We analysed the polymorphism of new loci using a set of 192 samples from 21 populations. Overall, all new markers (extended class I and class II) were less polymorphic, as indicated by their low mean PIC values (0.032–0.368) and mean H_o_ values (0.000–0.276). For Set 1, the MHC-S5 marker had the highest allele number, whereas the MHC-S2 marker had the lowest allele number. All alleles observed for MHC-S2 were homozygous in all populations, and therefore zero heterozygosity is reported.

A different Na, ranging from 4 to 38, was reported for each marker in Set 2 (Table 1). The marker LEI0258 was the most polymorphic in all populations (38 different alleles, H_o_ = 0.782, PIC = 0.912), whereas the marker MCW0312 was the least polymorphic (four alleles, H_o_ = 0.379, PIC = 0.406).

The allele frequency distribution for Set 2 markers varied by population. The 234- and 238-bp alleles of MHC-T; 214- and 218-bp alleles of MCW312; 307-, 310-, 313-, and 316-bp alleles of MHC-D; 193-, 249-, and 309-bp alleles of LEI0258; 202-, 205-, and 206-bp alleles of MCW0371; and 174-, 177-, 178-, and 179-bp alleles of MCW0370 had a high allele frequency above 10%. The MCW0371 and MCW0370 markers had higher allele numbers in all populations. However, MHC-T, MCW0371, and MCW0370 tended to have a null allele frequency, and there were low PIC values for MHC-T in many populations. A significant deviation from HWE was observed for these three markers in all populations except RIR. The F_IS_ values for each locus ranged from −0.197 (MHC-S4) to 1.00 (MHC-S2), which indicates excessive heterozygosity. Eight out of the 11 loci showed a significant departure from HWE, whereas only two loci could not be estimated because of zero or low H_o_. Eight private alleles (three in Korean chicken, two in Sri Lankan chicken, and one each in broiler and WL and HI) were observed for LEI0258, whereas no private alleles were reported for the other loci.

### 3.2. MHC Diversity within and between Populations

The allele distribution and the respective allele diversity statistics for both marker sets in each population according to origin are summarised in Table 2 and Table 3 and Supplementary Table S4. All populations had a similar level of diversity for Set 1 markers; however, there were differences in allele fixation by population.

For Set 2 markers, each population had a clear difference in MHC diversity. The mean Na, mean Ne, mean AR, and mean H_o_ of populations over six MS markers ranged from 2.33 (RIR, WL) to 8.17 (TA), 1.68 (WL) to 5.10 (KA), 2.30 (RIR) to 5.43 (KA), and 0.299 (JF) to 0.689 (CC1).

The Sri Lankan TA population was the most diverse, with a total of 49 alleles (mean allele number = 8.17), AR = 5.06, H_o_ = 0.495, and H_e_ = 0.657; the standard WL population was the least diverse, with a total of 14 alleles (mean allele number = 2.33), AR = 2.31, H_o_ = 0.265, and H_e_ = 0.342 (Table 4 and Supplementary Table S4). The Nigerian local population had similar MHC diversity to that of the Sri Lankan chicken. All Bangladeshi indigenous populations had higher diversity for all Set 2 markers except for two (MHC-T and MCW0370). The HI population had a high Na, AR, and H_o_; the JF population was an exception, with low diversity compared to the other Bangladeshi populations. All Korean populations had similar MHC diversity, but the hybrid YCC population was more diverse than the other hybrid populations.

The distribution of allele frequencies in Set 2 markers varied by population. The MCW0312 locus in RIR and WL and MCW0370 locus in WL had zero heterozygosity. The number of different alleles with a frequency less than 5% (rare alleles) was high in the indigenous populations from Sri Lanka, Bangladesh, and Nigeria compared to the commercial populations and Korean conserved populations. In the TA population, 50% of all alleles had a frequency of less than 5%, whereas all alleles identified in RIR, WL, and CC3 had >5% frequency.

#### Population Structure and Genetic Distances

The AMOVA results for the different population groups by country of origin (seven groups) demonstrated that most of the MHC molecular variation was distributed within populations (88.47%) rather than among populations (8.28%; *p* < 0.001; Table 4). This suggests that there was some genetic structure within the groups. We estimated the genetic distance and population differentiation at the MHC using five polymorphic loci with low null allele frequencies and high PIC values (PIC > 0.5) to avoid overestimating the population differentiation coefficient. The fixation index among groups was significant (F_CT_ = 0.033, *p* = 0.08), whereas, among populations, the within-group F_SC_ was 0.086 (*p* = 0.000), which indicates that populations were structured within at least one group.

The most closely related populations based on F_ST_ were Ross and Indian River and CC2 and CC3 (F_ST_ = 0.013, 0.011), whereas the WL and NW populations were the most unrelated (F_ST_ = 0.335). The Nei unbiased genetic distance (Nei D) and genetic identity value (Nei I) were consistent with the F_ST_ values: WL and NW had the lowest genetic identity and the highest genetic distance (uNei D = 1.321, Nei I = 0.267). The WL population had relatively large genetic distance (Nei D, 0.426–1.321) and F_ST_ values (0.215–0.385) compared to the other populations.

**Figure 2:**
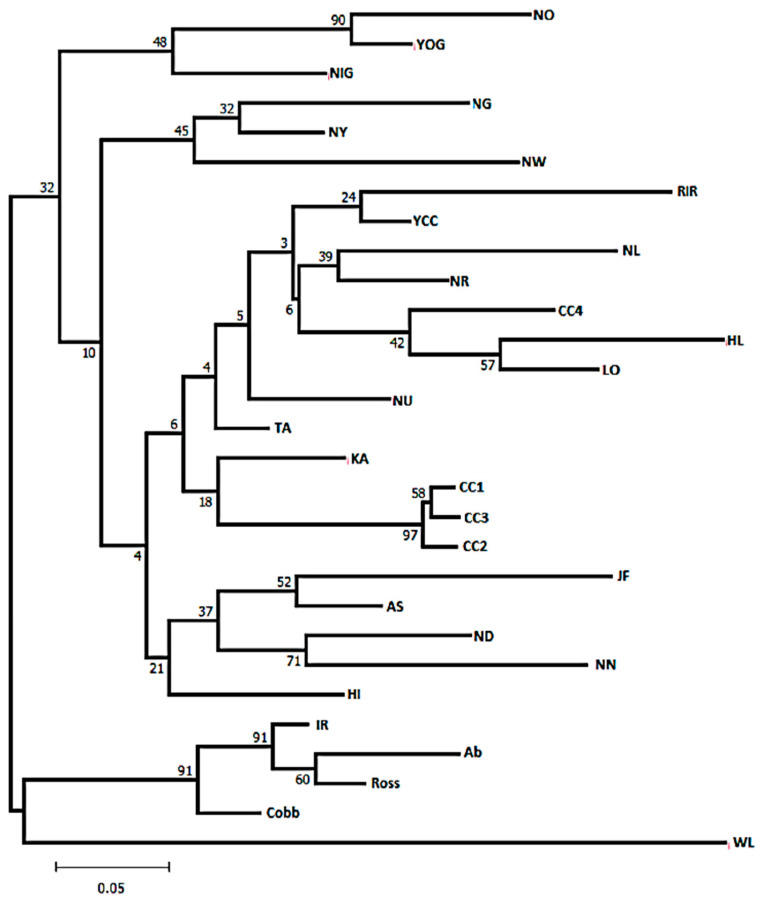
The Neighbour-joining tree of 29 populations constructed based on D_A_ distance using Set 2 MHC-*B* microsatellite markers. The population abbreviations are defined in Supplementary Table S2.

The NJ trees obtained using the DA distance revealed two main clusters, in which all broiler and WL populations were separated from all other populations (Figure 2). An imported and adapted RIR population sampled at the NIAS was grouped with the Korean conserved populations, in particular with YCC. Three Korean commercial hybrid populations (CC1, CC2, and CC3) always shared one separate cluster. Similarly, all commercial layer groups formed a separate cluster with the Korean CC4 population. No separate cluster for Sri Lankan chickens was noted, but these chickens did lie in between Korean and Bangladeshi chickens, with low bootstrap support. It is interesting that all Bangladeshi chickens had one separate cluster, in which JF had a close relationship with AS, whereas the ND and NN populations were grouped in a subclade of the main cluster.

### 3.3. Allele Distribution by Production Systems

**Figure 3:**
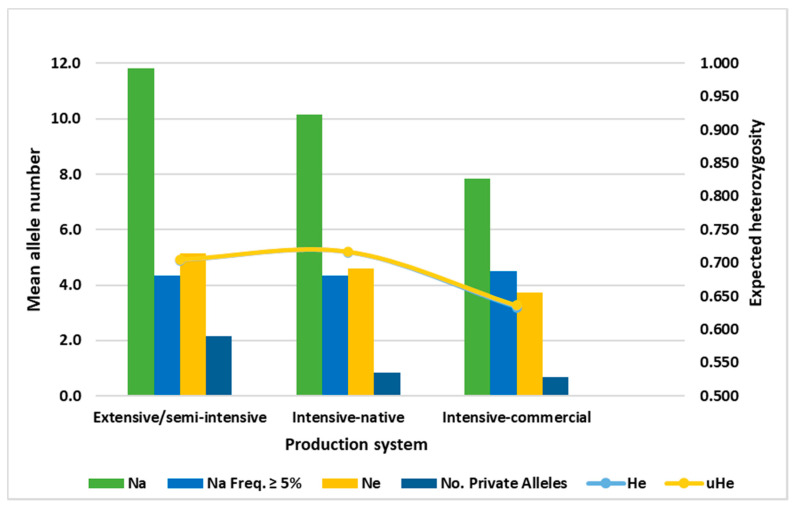
Mean allelic patterns of Set 2 microsatellite markers across the populations grouped based on their production systems. Na: number of different alleles; Ne: mean number of effective alleles; Na. Freq. ≥ 5%: number of alleles with more than 5% allele frequency; H_e_: expected heterozygosity and uH_e_: unbiased expected heterozygosity.

Clear differences in the mean allele number and AR were observed between populations under different farming systems (Table 5, Figure 3). The backyard or semi-intensive production groups had a higher mean allele number (11.17) and AR (4.46) than the intensive groups. However, independent t-tests of the mean differences in allele number and AR averaged over six loci showed no significant differences between the two production systems and the commercial group (*p* > 0.05; Table 5).

### 3.4. Haplotype Diversity

We constructed the MHC extended class I haplotypes using the Set 2 markers. The number of non-zero haplotypes constructed with the maximum likelihood method ranged from 4 (RIR) to 46 (TA; Table 6). The local populations of Sri Lankan and Bangladeshi chickens had a high number of unique haplotypes with a low haplotype frequency (<5%). A total of 89 haplotypes were shared. Commercial broiler and layer populations and Korean commercial hybrid populations had a higher number of shared haplotypes (Supplementary Table S5). Several haplotypes (22) were shared only among the Korean chickens. The four haplotypes identified in RIR were shared with other populations. The MHC-B_MS7 (similar to B21), and MHC-B_MS10 haplotypes had >5% frequency in the shared populations, except in a few populations, in which they had a low frequency of <5% (Supplementary Table S5). Thirteen haplotypes were identified in the WL population, but only two of them were shared. A comparison of all haplotypes identified for seven B standards (B2, B5, B12, B13, B15, B19, and B21) showed that the MHC-B_MS1(B2), MHC-B_MS2 (B15), MHC-B_MS4 (B13), and MHC-B_MS5 (B19) haplotypes in two populations, MHC-B_MS3 (B5) haplotype in three populations, and B21-like haplotype, MHC-B_MS7, was present in all 15 populations.

## 4. Discussion

Breeds from different environments, from different production systems, and most importantly with different breeding histories can have different genetic diversity [42]. These differences can be assessed with neutral and non-neutral genetic markers [43]. The MS markers in the MHC region provide a very useful and easy method for studying the genetic diversity of diverse populations.

### 4.1. Polymorphism of MHC-B Microsatellite Loci

A high number of polymorphic MS markers have been reported in the MHC class I, II, and III genes of humans, swine, and mice, whereas only six MS markers have been reported in the chicken MHC-*B* region. The minimal essential gene region of the chicken MHC is less complex, and some of the genes in the HLA and SLA regions are not reported in chicken, which might account for the low number of polymorphic MS markers in chicken. However, the available full sequence information for GGA16 is still inadequate for investigating probable MS markers. Among the reported markers, only the most polymorphic locus, LEI0258, has been used extensively in MHC studies of chicken breeds [22,25,44]. Other MS markers have not been used to investigate MHC diversity in many chicken breeds.

In this study, we evaluated two sets of MS markers. The polymorphism of new markers in Set 1 was assessed with 192 samples of 21 unrelated populations, and all loci had low PIC values (PIC < 0.5) and heterozygosity. Two markers, identified in the *Blec2* and *DMB1* genes, respectively, had little allele variation and low H_o_. This might be a consequence of the low variation in the *Blec2* and *DMB1* genes, as reported by Hosomichi et al. [33]. Five of the seven B standards used in this study had sizes of 220 and 261 bp, whereas the remaining two had 220 and 270 bp alleles for MHC-S4 and MHC-S5, respectively. The 14 sequences available from Hosomichi et al. [33] for other B standard haplotypes agreed with the current results in terms of allele sizes. However, we could not provide any evidence of variation for the first three markers (MHC-S1 to MHC-S3) in the aforementioned MHC-*B* haplotypes because of incomplete sequence information. We hypothesise that the intron and intergenic regions, where these three markers have been identified, may be less diverse.

According to the available 245 kb of complete MHC-*B* sequence information (GenBank accession no. AB2268588), the MHC0371 and MHC0370 loci contained mononucleotide repeats ([A]n) motifs and were located in the intron region of the *B-BTN2* and *BG1* genes. The MHC-T and MCW0312 markers contained (GG)n and (CA)n repeats, respectively, and were 7.9 kb apart from each other and approximately 50 kb upstream of the LEI0258 locus. The MHC-D marker was a tri-repeat motif of (TAA)n that was mapped in between LEI0258 and MCW0312 (11.8 kb from the MCW0312 marker). Because these six MS markers were located in the extended class I region, close to the class II region, there was a high linkage disequilibrium among them. The Set 2 markers had a high mean PIC and H_o_ compared to the novel loci. The high allele variation and PIC for LEI0258 concurs with the results of previous studies. The mean PIC values of these six loci (0.4–0.92) are comparable to those of the 68 markers in humans (0.6–0.7) and the 40 markers in the entire porcine SLA region (0.71–0.68) [16,17,18].

A high number of polymorphic markers in the extended class I region is considered likely because this region contains highly polymorphic genes, such as the tripartite 7 motif (*TRIM* genes), zinc finger protein gene, *HEP21,* guanine nucleotide-binding, and *BG* genes. The *B-TN1* and *B-TN2* genes, in which the LEI0258 and MCW0371 markers were identified, belong to the butyrophilin-like (*BTN*) gene cluster. The *TRIM-B30.2*-like domain likely helps regulate innate and adaptive immunity in higher vertebrates [11]. Duplication and shuffling events between *BG*-like and *B32.2*-like genes might be responsible for the formation of *BTN* genes in chicken. Therefore, we should expect substantial variation in the MS markers found in this gene region.

The occurrence of null alleles is more common in MSs because of mutations in the primer binding region or PCR amplification bias [45]. According to Gray [46], MS mutations are mainly due to DNA slippage during replication, whereas for a variable number tandem repeat marker the predominant mutation mechanism appears to be gene conversion and unequal crossing over. Therefore, we speculate that a high null allele frequency was reported for three loci—MHC-T, MCW0370, and MCW0371—in almost all populations for one of these reasons.

From the results of previous studies and sequences obtained in this study, it is very clear that insertion–deletion, single nucleotide polymorphism (SNP), and recombination are responsible for the evolution of different fragment sizes of LEI0258 loci. In line with Fulton et al. [22], we observed no PCR amplification for the MCW0370 locus for B5 and B15 standard samples, and as a result we reported a null allele for this locus. The assessment of two sequences of this B haplotype (AB426142 and AB426149) showed the absence of a primer binding region and repeat motifs of this marker in the B5 and 15 haplotypes. A similar problem of no PCR amplification or the presence of a null allele was detected in several individuals among the local chickens, and it is likely that these animals might have had mutations at these loci.

### 4.2. Difference in MHC Diversity and Frequency among Populations

Sri Lankan chickens had the highest MHC diversity among the South Asian breeds. However, the diversity was lower than that reported in several previous studies of African and Iranian local chickens compared using only the LEI0258 loci [27,44]. This higher diversity in Sri Lankan chickens is obvious, with each subpopulation composed of highly crossbred, mixed genotypes and with an absence of population structure as described in Silva et al. [47]. The lack of tightly controlled breeding practices under the backyard/semi-intensive production system allows interbreeding among different ecotypes and possible gene flow through the sharing of animals between households. This can result in a higher number of heterozygous animals and high within-population variation. A similar high MHC diversity in the same populations was observed using 90 SNPs in the MHC-*B* regions (unpublished data [48]).

The same population of Bangladeshi chickens was previously studied using 15 neutral markers [28] and low MHC diversity was reported, which suggests a balancing selection of MHC in these populations. This might reflect in part the influence of selection and breeding programs implemented in the recent past. However, the low MHC diversity in JF is probably associated with genetic drift and a small, fragmented population size.

Korean populations had consistent allele diversity, which is in agreement with our previous studies and explained the MHC diversity in Korean chicken breeds using the MHC-*B* SNP panel [30,49]. Compared to the YOG populations and Korean commercial hybrid populations, the six conserved populations had low diversity. These differences in attributes may have to do with selection and within line-breeding strategies implemented during their development [50]. As expected, three of the Korean commercial hybrids (CC1, CC2, and CC3) were diverse and shared a similar allele distribution because they shared the same parental lines, whereas CC4 was a semi-broiler chicken developed by crossing the parental lines of a layer and broiler. Despite having many shared alleles with the layers and broilers, the CC4 population was genetically closer to the HL population.

Allele richness is more sensitive than heterozygosity to founder events followed by population expansions [51]. The loss of alleles during founder events reduces the AR but not heterozygosity. This founder effect was obvious in the Korean Ogye population. A NIAS conserved population (NO) that had been separated from its original population (YOG) more than 20 years ago lost some of its alleles. For example, the 217-, 245-, 283-, 307-, and 465-bp alleles of LEI0258 that were observed in the original YOG population were not found in the NO population.

We noticed a high number of least frequent alleles in each population, except for the RIR, WL, and LO breeds. Alleles that occur with a frequency of <5% are considered rare and require much attention, as they can easily be wiped out from a population that has a low effective population size due to simple genetic drift. Most of the alleles were shared among the populations, which indicates that these alleles were dispersed into a wide range of native chicken breeds with various frequencies. The presence of many alleles and their frequencies is also of significance in response to selection [52,53]. Because MHC diversity is maintained by the host–pathogen interaction, the presence of many alleles can be helpful for counteracting the effects of mutations that occur in pathogen antigens invading host immunity [54]. The presence of rare alleles (low frequency) might be helpful for inducing an immune response against new pathogens when common alleles become the target of pathogen adaptation and are no longer resistant to these new pathogens [54,55].

### 4.3. Differences in MHC Diversity among Production Systems

Bettridge et al. [12] explained the role of local adaptation in the production of local village chickens. Regional variation in trait preferences and parasite burdens is associated with distinct chicken gene pools and almost certainly occurs in response to interactions between natural and human-driven selection pressures (e.g., consumer demand and breeding for improved traits). In this study, MHC diversity decreased with intensification (backyard extensive/semi-intensive > intensive > commercial). This might be a direct consequence of the selection of animals for genetic improvement (reduced effective population size). However, unlike commercial chickens that are reared by intensive farming, the exposure of local chickens to dynamic environmental variation and pathogens during their lifetime determines the survival of MHC alleles in these populations. Therefore, these observed differences might be associated with system adaptation, agro-ecology, parasite populations, and bird genotypes.

### 4.4. Haplotype Diversity

MS markers allow an evaluation of diversity not only at a single locus but also among haplotypes (i.e., unique combinations of alleles at each locus), which makes these assessments more comprehensive and reliable. The number of polymorphic MS markers in chicken is still limited compared to the number in humans, swine, and other livestock species [16,17,18,56]. There is large variation in the number of haplotypes identified in 29 populations. One possible reason for this difference might be that the different alleles of each marker are fixed differently in each population. If a population consists of a diverse range of individuals, it tends to have many haplotypes, as we noticed in the Sri Lankan, Bangladeshi, and Nigerian chickens. Local crossbred and commercial hybrid populations also had more total haplotypes and shared many of them with their related parental populations (Supplementary Table S5), which indicates that a possible population admixture occurred during their development. RIR chickens are standard brown egg layers reared in many countries. Their contribution to MHC diversity in other local indigenous populations or crossbreds was very high, which agrees with our previous studies and Fulton et al. [49,57]. The WL population is usually maintained for white egg production and less common in crossbreeding programs. Therefore, we would expect a smaller number of shared haplotypes between WL and other chicken breeds. The RIR and WL populations used in this study had low diversity mainly because of their small population size and closed breeding practices implemented at the NIAS.

The observed allele combinations of seven MHC-*B* standard haplotypes of the ADOL line were consistent with their original sizes reported by Fulton et al. [21] and the six homozygous MHC-*B* haplotypes described by Gao et al. [58]. The haplotype MHC-B_MS7, which corresponds to the B21 standard haplotype, was observed in 15 populations. The B21 haplotype, which is highly resistant to Marek’s disease, had a high allele frequency in the RIR, Cobb, IR, and NN populations and all Korean commercial hybrids but a low frequency in the AS, KA, TA, Ross, and Ab populations. Despite the differences in frequency, the segregation of this haplotype in wider populations is a sign of resistance to Marek’s disease in these populations.

The populations with a close genetic relationship shared many haplotypes. As revealed by the NJ trees, populations had a close genetic relationship when they shared either parental lines or past introgression events. However, some of the shared haplotypes were observed in genetically distant populations, which indicates that they may be equally important for resistance to disease in chickens.

## 5. Conclusions

Eleven MS markers, six previously developed markers, four new markers in the *TRIM/Blec* gene region, and one new marker in the class II region, displayed different polymorphism. Set 2 markers, in particular LEI0258, MCW0370, and MCW0371, had high allele diversity and high MHC haplotype diversity that varied across countries and production systems. These results indicate that each population has unique MHC diversity. The use of highly polymorphic markers would be very useful for reliable studies of MHC diversity and inference of disease resistance in chickens.

## Figures and Tables

**Table 1:** Summary of 11 microsatellite markers used in this study.

Marker	Range	N	Na	H_o_	H_e_	PIC	F_IS_	F (null)	HWE
MHC-S1 ^a^	231–263	166	4	0.205	0.402	0.368	0.380	0.313	***
MHC-S2 ^a^	227–233	181	3	0.000	0.033	0.032	1.000	0.443	nd
MHC-S3 ^a^	205–208	176	2	0.216	0.326	0.272	0.043	0.202	***
MHC-S4 ^a^	214–223	186	4	0.081	0.094	0.092	−0.197	0.093	nd
MHC-S5 ^a^	255–270	185	5	0.276	0.315	0.277	−0.027	0.064	ns
MHC-T ^b^	232–240	768	5	0.362	0.517	0.451	0.087	0.160	***
MCW0312 ^b^	210–218	867	4	0.379	0.494	0.406	−0.055	0.134	***
MHC-D ^b^	307–319	870	5	0.632	0.742	0.694	−0.046	0.079	***
LEI0258 ^b^	193–539	879	38	0.782	0.918	0.912	−0.034	0.080	***
MCW0371 ^b^	200–210	877	10	0.429	0.808	0.784	0.371	0.313	***
MCW0370 ^b^	168–182	816	12	0.308	0.837	0.818	0.525	0.465	***

^a^ Novel microsatellite markers identified in this study (Set 1); ^b^ microsatellite markers found in the literature (Set 2); N: number of samples; Na: number of identified alleles; H_o_: observed heterozygosity; H_e_: expected heterozygosity; PIC: polymorphic information content; F_IS_: MHC-linked microsatellite locus inbreeding coefficient; F (null): null allele frequency; HWE: a departure from the Hardy–Weinberg equilibrium (significance with Bonferroni correction; *** *p* < 0.0001; nd: not detected; ns: not significant).

**Table 2:** Mean allele number, allele richness, observed and expected heterozygosity values for Set 1 microsatellite markers in 21 populations.

Pop Code ^1^	Na	Ne	AR	H_o_	H_e_	F
KNC	1.8	1.23	1.32	0.104	0.165	0.316
YO	1.8	1.39	1.72	0.246	0.207	−0.175
YCC	1.8	1.16	1.56	0.107	0.115	0.036
CC1	1.6	1.22	1.49	0.125	0.142	0.077
CC2	1.4	1.24	1.39	0.100	0.150	0.166
CC3	1.8	1.45	1.67	0.171	0.204	0.220
CC4	1.4	1.34	1.38	0.075	0.127	0.135
KA	1.8	1.34	1.66	0.175	0.211	0.123
TA	2.0	1.47	1.82	0.159	0.238	0.284
ND	2.0	1.63	1.83	0.300	0.305	−0.006
NN	1.4	1.29	1.39	0.104	0.166	0.407
AS	2.0	1.56	1.91	0.425	0.345	−0.103
HI	2.0	1.43	1.76	0.232	0.240	0.009
JF	1.8	1.27	1.80	0.182	0.191	−0.011
ADOL	1.4	1.07	1.76	0.062	0.057	−0.084
RIR	1.6	1.36	1.59	0.233	0.225	−0.037
WL	1.4	1.37	1.40	0.219	0.192	−0.133
HY	1.6	1.19	1.76	0.123	0.132	0.373
IR	1.8	1.52	1.53	0.000	0.278	1.000
Ross	1.2	1.09	1.18	0.025	0.061	0.590
Ab	1.4	1.16	1.36	0.086	0.116	0.364
Mean	1.67	1.34		0.154	0.184	0.147

^1^ Population abbreviations are defined in Supplementary Table S2; Na = mean number of different alleles; Ne = mean number of effective alleles (Ne: 1/(1 − He); AR: mean allele richness per population estimated based on the minimum number of sample size; according to Kalinowsky [38]; H_o_: observed heterozygosity; H_e_: expected heterozygosity; F: fixation index (1 − (H_o_/H_e_)).

**Table 3:** Mean allele number, allele richness, observed and expected heterozygosity values for Set 2 microsatellite markers in 29 populations.

Pop Code ^1^	N	Na	Ne	AR	H_o_	H_e_	F	F_IS_
NG	49.67	3.67	2.30	2.98	0.390	0.485	0.204	0.0585
NL	49.67	3.67	2.38	2.87	0.523	0.546	0.020	−0.0390
NR	43.17	4.50	3.10	3.75	0.508	0.587	0.162	0.0307
NW	47.17	5.00	2.56	3.73	0.463	0.560	0.146	0.1348
NY	50.00	4.33	3.31	3.82	0.574	0.639	0.054	0.1193
NO	45.00	3.83	2.05	2.94	0.338	0.468	0.378	0.0682
YO	59.33	5.17	2.81	3.79	0.409	0.582	0.293	0.1242
YCC	47.83	5.83	3.10	4.00	0.553	0.612	0.081	0.0114
CC1	19.83	3.83	2.79	3.42	0.689	0.615	−0.114	−0.1023
CC2	20.00	4.33	2.76	3.63	0.475	0.596	0.190	0.1849
CC3	19.00	4.00	3.17	3.71	0.569	0.644	0.102	0.0415
CC4	20.00	4.33	2.99	3.86	0.525	0.561	0.018	−0.0689
KA	18.83	6.50	5.10	5.43	0.652	0.735	0.067	0.0992
NU	17.83	6.33	4.04	5.02	0.447	0.650	0.306	0.1983
TA	34.17	8.17	4.19	4.96	0.495	0.657	0.259	0.1559
ND	36.00	5.50	3.22	4.05	0.469	0.650	0.264	0.1396
NN	45.17	5.17	2.26	3.29	0.364	0.531	0.309	0.2137
AS	16.83	5.83	3.79	4.90	0.511	0.704	0.277	0.1979
HI	38.17	6.33	3.99	4.86	0.594	0.679	0.148	0.0866
JF	18.33	3.67	2.56	3.32	0.299	0.530	0.408	0.3778
NIG	18.50	6.00	3.73	4.77	0.507	0.645	0.206	0.0145
RIR	14.00	2.33	1.86	2.30	0.452	0.412	−0.109	−0.1513
WL	15.50	2.33	1.68	2.31	0.265	0.342	0.345	0.1266
HY	18.67	3.50	2.02	2.91	0.339	0.421	0.081	0.0623
LO	19.17	3.00	2.31	2.80	0.429	0.495	0.060	−0.0913
IR	15.67	4.50	3.12	3.97	0.631	0.614	−0.056	−0.0126
Ross	15.67	4.83	2.57	3.74	0.551	0.533	−0.024	−0.0334
Ab	16.67	4.00	2.25	3.27	0.468	0.531	0.101	0.12057
Cobb	16.33	4.50	3.14	3.92	0.581	0.620	0.021	0.11709

^1^ Populations abbreviations are defined in Supplementary Table S2; N: mean number of samples; Na: mean number of different alleles; Ne: mean number of effective alleles (Ne: 1/(1 − He); AR: mean allele richness per population estimated based on the minimum number of sample size according to Kalinowsky [38]; H_o_: observed heterozygosity; H_e:_ expected heterozygosity; F: fixation index (heterozygosity deficient (1 − (Ho/He)); F_IS_: populations specific F_IS_ (1023 permutations).

**Table 4:** Analysis of molecular variance (AMOVA) by locus, groups based on the country of origin using Set 2 microsatellite markers.

Source of Variation ^1^	d.f.	Variance Component	% Variance	Fixation Index	Significance (*p*-Value)
Among groups	6	219.22	3.26	F_ST_ = 0.115	0.000
Among populations within groups	22	557.33	8.28	F_SC_ = 0.086	0.000
Within populations	1473	5955.95	88.47	F_CT_ = 0.033	0.081

^1^ Variance components were calculated using only five polymorphic loci in the Set 2 microsatellite markers; d.f.: degree of freedom; F_ST:_ proportion of genetic variation within populations, F_SC_: variation among populations within groups, F_CT_: variation among groups (seven groups were set based on their origin).

**Table 5:** Allele number and allele richness and unbiased expected heterozygosity difference for Set 2 MS markers in chicken populations grouped by their production systems.

Genetic Diversity					
Production System ^1^	Marker	N ^2^	Number of Alleles	Allele Richness ^3^	uH_e_
Backyard/Semi-intensive system	MHC-T	291	4	2.79	0.521
	MHC0312	314	4	2.68	0.459
	LEI0258	316	33	7.88	0.926
	MHC-D	315	5	3.71	0.741
	MCW0371	315	10	5.05	0.766
	MCW0370	308	11	4.62	0.817
^4^ Mean (SE)		309.83	11.17 (4.54)	4.46 (0.79)	0.705
	Marker	N	Number of alleles	Allele richness	uH_e_
Intensive system for native chicken	MHC-T	376	5	2.05	0.512
	MHC0312	453	4	2.04	0.527
	LEI0258	459	26	4.94	0.884
	MHC-D	454	5	3.41	0.736
	MCW0371	458	10	3.76	0.814
	MCW0370	412	11	3.79	0.828
^4^ Mean (SE)		435.3	10.17 (3.38)	3.33 (0.46)	0.717
	Marker	N	Number of alleles	Allele richness	uH_e_
Intensive system for commercial chicken	MHC-T	101	3	1.90	0.468
	MHC0312	100	2	1.97	0.249
	LEI0258	104	18	5.46	0.861
	MHC-D	101	4	3.24	0.674
	MCW0371	104	8	3.83	0.774
	MCW0370	96	10	4.23	0.790
^4^ Mean (SE)		101.00	7.5 (2.45)	3.44(0.56)	0.636

^1^ Production systems are as defined in Supplementary Table S1; ^2^ number of samples; ^3^ allele richness per population estimated based on the minimum number of sample size; ^4^ mean value with standard deviation in brackets; uHe: unbiased Expected Heterozygosity = (2N / (2N − 1)) × He.

**Table 6:** Number of MHC-*B* haplotypes identified using Set 2 microsatellite markers.

Pop Code ^1^	Total N. ha ^2^	N ha ^3^ (F > 5%)	Unique Haplotypes ^4^
RIR	4	4	0
WL	13	9	11
NG	40	4	29
NL	22	4	13
NR	17	9	5
NW	17	5	7
NY	22	7	4
NO	13	5	4
YOG	34	5	20
HI	32	3	21
ND	36	3	24
NN	18	4	11
AS	26	3	22
JF	14	7	13
KA	31	4	19
NU	24	8	19
TA	46	1	30
NIG	20	7	14
YCC	27	5	7
CC1	14	6	3
CC2	15	7	5
CC3	16	8	3
CC4	19	8	7
IN	14	6	4
Ab	13	8	4
Cobb	15	6	7
Ross	19	4	6
HL	13	6	6
LO	12	6	1

^1^ Populations abbreviations are defined in Supplementary Table S2; ^2^ total number of haplotypes identified; ^3^ number of different haplotypes in each population with >5% haplotype frequencies; ^4^ haplotypes that observed only in one population.

## Data Availability

The data presented in this study are available on request from corresponding author.

## Supplementary Materials

The following are available online at https://www.mdpi.com/2073-4425/12/2/240/s1, Table S1: Characteristics of the farming systems described in the present study populations, Table S2: Characteristics of populations used in this study, Table S3: Microsatellite markers identified in MHC-*B* regions, allele size, and PCR primer information, Table S4: Summary of genetic diversity of MHC-B microsatellite markers (Set 2) in 29 populations, Table S5: Haplotypes using 6 microsatellite markers for 29 populations (Haplotype frequency >5%); Figure S1. Agarose gel results for the allele size distribution of Set 1 markers (MHC-S1~ S5) and Set 2 (MHC-T, MHC-D, MCW0312, MCW0370, MCW0371, LEI0258) markers in 7 control samples of known MHC-B haplotypes (from ADOL line) and negative samples (NC).
