# Supplementary material for: High Levels of Genetic Variation in MHC-Linked Microsatellite Markers from Native Chicken Breeds
Source: Genes (Basel). 2021 Feb 8;12(2):240. doi: 10.3390/genes12020240 (PMC7915948; doi:10.3390/genes12020240)
Supplement: Supplementary file 1 [file genes-12-00240-s001.pdf]

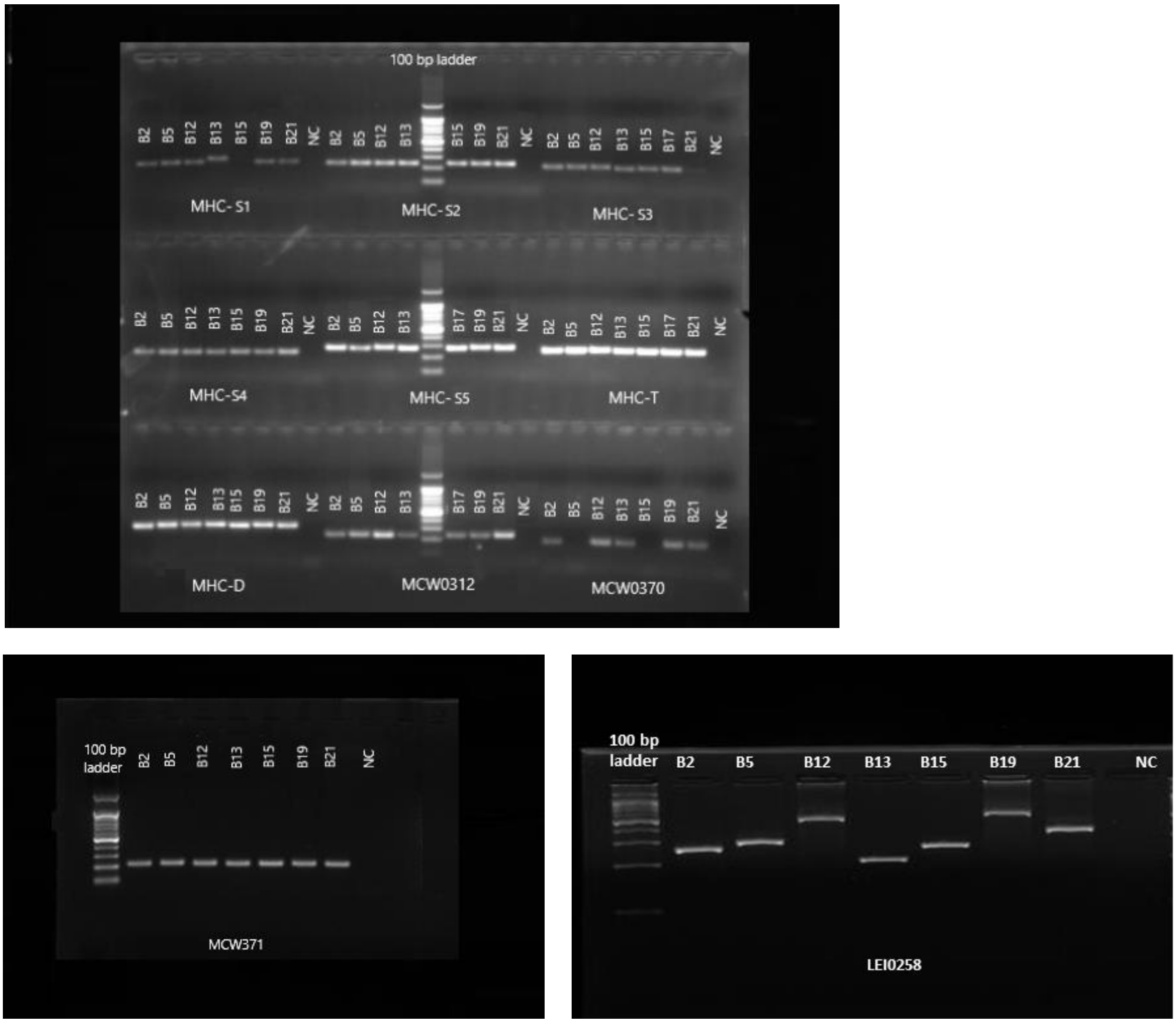

Figure S1. Agarose gel results for the allele size distribution of Set 1 markers (MHC-S1~ S5) and Set 2 (MHC-T, MHC-D, MCW0312, MCW0370, MCW0371, LEI0258) markers in 7 control samples of known MHC-B haplotypes (from ADOL line) and negative samples (NC).

Table S1. Characteristics of the farming systems described in the present study populations.

Criteria	Backyard scavenging	Semi-Intensive	Intensive (for native chicken breeds)	Intensive (for Commercial chicken)
Flock size	2-10	5-30	>100	>1000
Breeds	indigenous eco-types	indigenous eco-types/ breeds	hybrids, Indigenous breeds	elite hybrids, standard breeds
Source of New chicks	natural incubation	natural incubation, purchase or sharing	artificial incubation	purchase from commercial hatchery
Breeding/ selection	no selection	seldom selection	intensive selection and breeding	intensive selection
Feed sources	scavenging zero of low occasional supplementation	scavenging, regular supplementation	formulated compound feeds	high quality formulated compound feeds and additives
Access to veterinary services	zero or rarely	rarely	high	very high
Mortality	>70 %	high >50	low	low
Disease occurrence	high	high	low	low
Biosecurity and supportive strategies for diseases control	minimum	minimum	high	very high

Table S2. Characteristics of populations used in this study.

Origin	Breed /ecotype	Breed code	Characteristics	N	Production system
Korea	KNC Gray brown	NG	Native restored line	50	[1] Intensive
	KNC Black	NL	Native restored line	50	[1] Intensive
	KNC Red brown	NR	Native restored line	46	[1] Intensive
	KNC Yellow brown	NY	Native restored line	53	[1] Intensive
	KNC White	NW	Native restored line	52	[1] Intensive
	KNC NIAS Ogye	NO	Indigenous black chicken	49	[1] Intensive
	KNC Yeonsan Ogye	YO	Indigenous black chicken	62	Semi-Intensive
	Commercial hybrid line 1(NIAS)	YCC	Native commercial hybrid	48	[1] Intensive
	Hanhyup hybrid line 1	CC1	Native commercial hybrid	20	[1] Intensive
	Hanhyup hybrid line 2	CC2	Native commercial hybrid	20	[1] Intensive
	Hanhyup hybrid line 3	CC3	Native commercial hybrid	20	[1] Intensive
	Hanhyup hybrid line 4	CC4	Semi broiler	20	[1] Intensive
Standard	Rhode Island Red	RIR	Imported and adapted	14	[1] Intensive
Standard	White Leghorn	WL	Imported and adapted	17	[1] Intensive
Sri Lanka	Kandy ecotype	KA	Indigenous chicken	19	Semi-intensive
	Udapussellawa ecotype	NU	Indigenous chicken	18	Backyard
	Karuwalgaswewa	TA	Indigenous chicken	35	Semi-intensive
Bangladesh	Aseel	AS	Indigenous chicken	18	Semi-intensive
	Hilly chicken	HI	Indigenous chicken	39	Semi-intensive
	Naked Neck	NN	Indigenous chicken	46	Semi-intensive
	Non-descript common deshi	ND	Indigenous chicken	41	Semi-intensive
	Red Jungle fowl	JF	Wild red jungle fowl	19	Wild collection
Nigeria	Umuobasi ecotype	NIG	Indigenous chicken	20	Backyard
Commercial broiler	Ross	Ross	Broiler	16	[2] Intensive
	Cobb	Cobb	Broiler	17	[2] Intensive
	Abor Acres	Ab	Broiler	17	[2] Intensive
	Indian River	IR	Broiler	16	[2] Intensive
Commercial layer	HY-Line	HY	Layer	19	[2] Intensive
	Loman Brown	LO	Layer	20	[2] Intensive
MHC- B standard	ADOL line	ADOL	Experimental flock, Avian Disease and Oncology Laboratory (ADOL)	7	

N: Number of animals; [1] intensive system practices for native breeds in Korea; [2] Intensive system practices for commercial layer and broiler breeds.

Table 3. Microsatellite markers identified in MHC-B regions, allele size, and PCR primer information.

Marker	Repeat unit	Repeat range	Allele Size (bp)	Physical location	Near gene	Forward primer (5'-3')	Reverse primer (5'-3)	Reference
MHC-S1 [1]	(ATAA)n	6	~239	2438352-2438591	Intron	AAGGCTCCCTCCAGGAAA	AGTGCTGGGCTGAAGCTG	New
MHC-S2 [1]	(GGA)n	11.7	~229	2463432-2463661	BZFP2	GAGAAACCCTTCCAATGTGC	GTTTGGGTCCCCTTATGCTT	New
MHC-S3 [1]	(AGG)n	25.7	~211	2468365/2468576	intergenic	TTTGCCCCTTTCCCTCTC	ACATTGGAGCTGAAGGCG	New
MHC-T [2]	(GG)n	16	~237	2484354-2484591	Trim 7.1	ATGGTGGCCAAGTAAACTGGAG	GGATCTGACAGCTGAGCGAGGT	Sironi *et al.* [23]
MCW0312 [2]	(CA)n	9	217-221	2492560-2492775	HEP21	TTTGTTCGGGATTAAGCTTGG	CCTAAATCAGGATGTTTGGAC	Crooijmans *et al.* [19]
MHC-D [2]	(TAA)n		307-317	2525815-2526125	GNB2L1	CTGTTGGCGTTACAGAGCT	TTCACCCAGCAGCCTCTATC	Sironi *et al.* [23] Gao *et al* [56]
LEI0258 [2]	(R13)n (R12)n	18.3	194-543	2537930-2538299	B-BTN1	CACGCAGCAGAACTTGGTAAGG	AGCTGTGCTCAGTCCTCAGTGC	Fulton *et al.* [22]
MCW0371 [2]	(A)n		199-211	2548375-2548578	B-BTN2	CTGCTCCGAGCTGTAATCCTG	TTTCATGGCATCCTAAGATGG	Fulton *et al.* [22]
MCW0370 [2]	(A)n		~179	2550447-2550627	BG1	AAAGAGGAGAGTAGTTCACG	CCCACCTCATCATGCATTCC	Fulton *et al.* [22]
MHC-S4 [1]	(CAG)n	9	~220	2559804-2560024	BLEC2	GAGCTTCGATTTCCTGTGCA	TGGAGGGGCTCTTTCACAG	New
MHC-S5 [1]	(GAG)n	13	~261	2586620-2586881	DMB1	GTGTCCTTGTTTGTCCCCAC	CACATCCTACCTGCTGTCCC	New

[1] Set 1 microsatellite markers reported in this study.[2] Set 2 microsatellite markers reported in the literature. Physical position as per *Gallus gallus 6.0a* assembly available in the NCBI data base.

Table S4. Summary of genetic diversity of MHC-B microsatellite markers (Set 2) in 29 populations.

Pop [1]	Locus	Range	N	Na	Ne	AR	Ho	He	PIC	F (null)	F	HWE
NG	MHC-T	234-238	50	2	1.150	1.72	0.140	0.130	0.122	-0.028	-0.075	ns
	MCW0312	214-218	50	2	1.445	1.98	0.260	0.308	0.260	0.084	0.155	ns
	MHC-D	307-316	50	4	3.021	3.29	0.720	0.669	0.601	-0.042	-0.076	ns
	LEI0258	193-443	50	7	3.876	5.07	0.820	0.742	0.707	-0.053	-0.105	ns
	MCW0371	202-209	50	5	2.458	3.83	0.400	0.593	0.586	0.226	0.361	***
-	MCW0370	178-179	48	2	1.882	2.00	0.000	0.469	0.359	0.999	1.000	***
NL	MHC-T	234-238	48	2	1.822	2.00	0.521	0.451	0.349	-0.072	-0.154	ns
	MCW0312	214-218	50	2	1.891	2.00	0.560	0.471	0.360	-0.086	-0.188	ns
	MHC-D	307-316	50	3	1.557	2.48	0.360	0.358	0.316	-0.156	-0.007	ns
	LEI0258	193-333	50	5	3.181	3.79	0.820	0.686	0.626	-0.095	-0.196	ns
	MCW0371	202-208	50	6	3.106	3.72	0.560	0.678	0.617	0.076	0.174	***
	MCW0370	172-178	50	4	2.713	3.26	0.320	0.631	0.561	0.324	0.493	***
NR	MHC-T	234-238	29	2	1.228	1.87	0.069	0.185	0.168	0.449	0.628	***
	MCW0312	214-218	46	2	1.628	2.00	0.478	0.386	0.311	-0.107	-0.240	ns
	MHC-D	307-316	46	4	3.258	3.54	0.587	0.693	0.630	0.083	0.153	ns
	LEI0258	193-443	46	7	4.046	5.00	0.739	0.753	0.714	0..0107	0.018	ns
	MCW0371	202-208	46	5	3.296	4.15	0.674	0.697	0.653	0.039	0.033	*
	MCW0370	172-181	46	7	5.174	5.93	0.500	0.807	0.781	0.237	0.380	***
NW	MHC-T	234-238	36	3	1.989	2.22	0.472	0.497	0.387	0.029	0.050	ns
	MCW0312	214-218	47	2	1.313	1.93	0.234	0.238	0.210	0.009	0.018	ns
	MHC-D	307-316	47	4	2.749	3.65	0.723	0.636	0.576	-0.066	-0.137	ns
	LEI0258	193-478	52	7	3.052	4.79	0.731	0.672	0.635	-0.040	-0.087	ns
	MCW0371	201-207	52	6	3.930	5.05	0.269	0.746	0.707	0.476	0.639	***
	MCW0370	171-181	49	8	2.339	4.76	0.347	0.572	0.550	0.301	0.394	***
NY	MHC-T	234-238	36	2	1.829	2.00	0.583	0.453	0.351	-0.125	-0.287	ns
	MCW0312	214-218	53	2	1.564	1.99	0.396	0.360	0.295	-0.047	-0.099	ns
	MHC-D	307-316	53	4	3.428	3.90	0.717	0.708	0.658	-0.011	-0.012	ns
	LEI0258	193-478	53	6	4.266	5.05	0.755	0.766	0.730	0.062	0.014	ns
	MCW0371	202-209	53	6	4.860	5.23	0.472	0.794	0.770	0.252	0.406	***
	MCW0370	172-179	52	6	3.905	4.73	0.519	0.744	0.702	0.181	0.302	***
NO	MHC-T	234-236	31	2	1.371	1.96	0.000	0.271	0.234	0.973	1.000	***
	MCW0312	214-218	49	2	1.350	1.95	0.184	0.259	0.226	0.171	0.292	*
	MHC-D	307-316	49	4	2.736	3.50	0.653	0.635	0.566	-0.026	-0.029	ns
	LEI0258	205-443	49	5	2.650	3.62	0.633	0.623	0.550	-0.009	-0.016	ns
	MCW0371	200-207	49	5	2.408	3.31	0.510	0.585	0.534	0.092	0.127	***
	MCW0370	171-179	43	5	1.771	3.30	0.047	0.435	0.405	0.811	0.893	***
YO	MHC-T	234-238	59	3	1.899	2.52	0.305	0.473	0.395	0.210	0.355	***
	MCW0312	214-218	60	2	1.301	1.91	0.200	0.231	0..204	0.072	0.135	ns
	MHC-D	307-316	61	4	2.883	3.70	0.574	0.653	0.600	0.056	0.122	*
	LEI0258	205-469	61	10	4.417	5.91	0.738	0.773	0.744	0.026	0.045	ns
	MCW0371	202-208	62	7	3.635	5.02	0.565	0.720	0.682	0.133	0.216	***
	MCW0370	175-179	53	5	2.806	3.71	0.075	0.644	0.583	0.787	0.883	***

[1] Populations abbreviations are defined in Table S2; N: number of sample; Na: number of different allele for each locus; Ne: effective allele size; AR: allele richness; H_o: observed heterozygosity; H_e:

expected heterozygosity; PIC: Polymorphic information content; F(null): null allele frequencies, F: fixation index (loss of heterozygosity = 1- (Ho/He)), HWE: deviations of Hardy-Weinberg equilibrium (* $p < 0.05$; *** $p < 0.001$; ns : not significant; nd : not detected).

Table S4. Summary of genetic diversity of MHC-*B* microsatellite markers (Set 2) in 29 populations (Continued).

Pop [1]	Locus	Range	N	Na	Ne	AR	Ho	He	PIC	F(null)	F	HWE
YCC	MHC-T	234-238	47	3	1.862	2.17	0.362	0.463	0.365	0.122	0.219	ns
	MCW0312	214-218	48	2	1.465	1.98	0.396	0.317	0.267	-0.108	-0.247	nd
	MHC-D	307-316	48	4	3.421	3.84	0.792	0.708	0.654	-0.059	-0.119	ns
	LEI0258	193-479	48	12	5.473	7.02	0.917	0.817	0.799	-0.060	-0.122	ns
	MCW0371	202-209	48	6	2.939	4.14	0.396	0.660	0.303	0.258	0.400	ns
	MCW0370	172-182	48	8	3.434	4.83	0.458	0.709	0.665	0.209	0.353	ns
CC1	MHC-T	234-238	19	2	1.994	2.00	0.737	0.499	0.374	-0.193	-0.478	nd
	MCW0312	210-218	20	3	2.156	2.96	0.600	0.536	0.477	-0.065	-0.119	nd
	MHC-D	307-316	20	4	3.008	3.40	0.950	0.668	0.601	-0.181	-0.423	ns
	LEI0258	217-443	20	6	4.348	5.18	1.000	0.770	0.735	-0.139	-0.299	ns
	MCW0371	201-206	20	4	2.241	3.59	0.300	0.554	0.509	0.294	0.458	***
	MCW0370	174-181	20	4	2.996	3.40	0.550	0.666	0.599	0.082	0.174	*
CC2	MHC-T	234-238	20	2	1.724	2.00	0.500	0.420	0.332	-0.087	-0.190	ns
	MCW0312	210-218	20	3	2.381	2.88	0.550	0.580	0.492	0.028	0.052	ns
	MHC-D	307-316	20	4	2.640	3.39	0.600	0.621	0.559	0.023	0.034	ns
	LEI0258	217-461	20	8	4.878	5.94	0.700	0.795	0.767	0.060	0.119	ns
	MCW0371	201-206	20	4	2.015	3.55	0.250	0.504	0.466	0.318	0.504	***
	MCW0370	174-181	20	5	2.909	4.03	0.250	0.656	0.605	0.459	0.619	***
CC3	MHC-T	234-238	17	2	1.778	2.00	0.529	0.438	0.342	-0.095	-0.209	ns
	MCW0312	210-218	19	3	2.481	2.95	0.684	0.597	0.516	-0.069	-0.146	ns
	MHC-D	307-316	19	4	3.209	3.67	0.737	0.688	0.627	-0.032	-0.070	ns
	LEI0258	217-457	20	7	5.594	6.24	0.900	0.821	0.798	-0.043	-0.096	ns
	MCW0371	201-206	20	4	2.564	3.61	0.300	0.610	0.554	0.326	0.508	***
	MCW0370	174-181	19	4	3.422	3.82	0.263	0.708	0.651	0.457	0.628	***
CC4	MHC-T	234-238	20	2	1.471	1.99	0.400	0.320	0.269	-0.110	-0.250	ns
	MCW0312	214-218	20	2	1.220	1.88	0.200	0.180	0.164	-0.046	-0.111	ns
	MHC-D	307-316	20	4	2.930	3.77	0.800	0.659	0.595	-0.101	-0.214	ns
	LEI0258	193-474	20	8	5.797	6.58	1.000	0.828	0.805	-0.099	-0.208	ns
	MCW0371	203-209	20	4	2.974	3.76	0.550	0.664	0.603	0.112	0.171	*
	MCW0370	172-179	20	6	3.556	5.20	0.200	0.719	0.683	0.570	0.722	***
RIR	MHC-T	232-238	14	3	2.240	2.83	0.500	0.554	0.460	0.063	0.097	ns
	MCW0312	218	14	1	1.000	1.00	0.000	0.000	nd	nd	nd	nd
	MHC-D	307-316	14	3	2.240	2.99	0.643	0.554	0.493	-0.059	-0.161	ns
	LEI0258	295-357	14	3	2.240	2.99	0.643	0.554	0.493	-0.059	-0.161	ns
	MCW0371	205-208	14	2	1.508	2.00	0.429	0.337	0.280	-0.119	-0.273	ns
	MCW0370	174-179	14	2	1.912	2.00	0.500	0.477	0.363	-0.024	-0.048	ns
WL	MHC-T	234-236	17	2	1.262	1.93	0.118	0.208	0.186	0.276	0.433	ns
	MCW0312	218	17	1	1.000	1.00	0.000	0.000	0.000	nd	nd	nd
	MHC-D	310-316	17	3	2.181	2.97	0.529	0.542	0.480	0.028	0.022	ns
	LEI0258	261-539	17	3	2.181	2.97	0.529	0.542	0.480	0.028	0.022	ns
	MCW0371	203-209	17	3	2.198	2.98	0.412	0.545	0.486	0.119	0.244	ns
	MCW0370	174-176	8	2	1.280	2.00	0.000	0.219	0.195	nd	1.000	**

[1] Populations abbreviations are defined in Table S2.

Table S4. Summary of genetic diversity of MHC-*B* microsatellite markers (Set 2) in 29 populations (continued).

Pop [1]	Locus	Range	N	Na	Ne	AR	Ho	He	PIC	F(null)	F	HWE
HI	MHC-T	234-238	34	3	1.487	2.68	0.206	0.327	0.303	0.216	0.371	**
	MCW0312	210-218	39	4	2.733	3.55	0.641	0.634	0.565	-0.014	-0.011	ns
	MHC-D	307-319	39	5	4.402	4.74	0.846	0.773	0.736	-0.047	-0.095	ns
	LEI0258	193-489	39	13	7.881	8.38	0.821	0.873	0.861	0.026	0.060	*
	MCW0371	200-208	39	6	3.803	4.89	0.487	0.737	0.695	0.219	0.339	***
	MCW0370	168-179	39	7	3.669	4.95	0.564	0.727	0.687	0.118	0.225	***
ND	MHC-T	232-238	38	4	2.498	3.33	0.474	0.600	0.538	0.106	0.210	**
	MCW0312	212-218	40	3	1.809	2.82	0.400	0.447	0.401	0.079	0.106	ns
	MHC-D	307-316	41	4	2.410	3.13	0.512	0.585	0.519	0.064	0.125	ns
	LEI0258	193-417	41	12	5.133	6.69	0.780	0.805	0.780	0.017	0.031	ns
	MCW0371	201-208	40	6	3.778	4.32	0.525	0.735	0.689	0.166	0.286	*
	MCW0370	168-179	16	4	3.683	3.98	0.125	0.729	0.680	0.711	0.828	***
NN	MHC-T	234-240	42	4	2.592	3.15	0.310	0.614	0.544	0.350	0.496	***
	MCW0312	210-218	46	4	2.134	2.49	0.457	0.531	0.421	0.068	0.141	***
	MHC-D	307-316	46	4	1.436	2.70	0.283	0.304	0.285	0.044	0.069	ns
	LEI0258	193-405	46	9	3.146	4.66	0.674	0.682	0.629	0.011	0.012	ns
	MCW0371	200-206	46	6	1.903	3.78	0.174	0.474	0.448	0.486	0.633	***
	MCW0370	168-179	45	4	2.368	2.94	0.289	0.578	0.487	0.325	0.500	***
AS	MHC-T	234-240	15	4	2.406	3.32	0.333	0.584	0.500	0.262	0.430	**
	MCW0312	212-218	18	3	2.656	3.00	0.556	0.623	0.553	0.084	0.109	**
	MHC-D	307-319	18	5	3.161	4.12	0.667	0.684	0.627	0.022	0.025	ns
	LEI0258	193-381	18	11	6.231	7.88	0.667	0.840	0.822	0.098	0.206	***
	MCW0371	200-206	18	6	4.985	5.59	0.556	0.799	0.771	0.154	0.305	***
	MCW0370	168-179	14	6	3.294	5.49	0.286	0.696	0.668	0.432	0.590	**
JF	MHC-T	234-236	15	2	1.471	1.99	0.267	0.320	0.269	0.091	0.167	ns
	MCW0312	212-218	19	2	1.430	1.99	0.263	0.301	0.255	0.066	0.124	ns
	MHC-D	307-316	19	3	2.235	2.82	0.368	0.553	0.464	0.218	0.333	ns
	LEI0258	193-381	19	6	4.457	5.41	0.579	0.776	0.743	0.145	0.254	**
	MCW0371	201-208	19	6	3.780	5.04	0.316	0.735	0.694	0.405	0.571	*
	MCW0370	177-179	19	3	1.994	2.67	0.000	0.499	0.416	1.000	1.000	***
KA	MHC-T	234-238	19	3	2.105	2.42	0.737	0.525	0.412	-0.171	-0.404	ns
	MCW0312	210-218	19	4	2.569	3.64	0.737	0.611	0.556	-0.091	-0.206	ns
	MHC-D	307-316	19	4	3.505	3.95	0.737	0.715	0.663	-0.032	-0.031	ns
	LEI0258	193-474	19	14	10.939	10.04	0.947	0.909	0.901	-0.024	-0.043	ns
	MCW0371	201-208	19	8	5.870	6.38	0.421	0.830	0.818	0.329	0.492	***
	MCW0370	174-181	18	7	5.635	6.17	0.333	0.823	0.799	0.424	0.595	***
NU	MHC-T	234-240	17	3	2.198	2.98	0.294	0.545	0.486	0.285	0.460	**
	MCW0312	214-218	18	2	1.456	1.99	0.278	0.313	0.264	0.060	0.113	ns
	MHC-D	307-316	18	4	3.100	3.88	0.722	0.677	0.062	-0.041	-0.066	ns
	LEI0258	193-443	18	16	9.969	10.39	0.889	0.900	0.892	0.004	0.012	ns

	MCW0371	201-208	18	7	3.600	5.78	0.278	0.722	0.696	0.451	0.615	***
	MCW0370	172-179	18	6	3.904	5.12	0.222	0.744	0.704	0.553	0.701	***
TA	MHC-T	234-240	32	4	1.725	2.50	0.313	0.420	0.355	0.142	0.257	ns
	MCW0312	210-218	34	4	1.724	2.96	0.294	0.420	0.377	0.162	0.300	**
	MHC-D	307-316	34	4	3.477	3.81	0.676	0.712	0.657	0.024	0.050	ns
	LEI0258	193-443	35	21	10.29	9.72	0.943	0.903	0.890	-0.025	-0.044	ns
	MCW0371	201-209	35	8	3.470	5.28	0.429	0.712	0.676	0.266	0.398	**
	MCW0370	168-180	35	8	4.463	5.48	0.314	0.776	0.742	0.425	0.595	***

[1] Populations abbreviations are defined in Table S2.

Table S4. Summary of genetic diversity of MHC-*B* microsatellite markers (Set 2) in 29 populations (continued).

Pop [1]	Locus	Range	N	Na	Ne	AR	Ho	He	PIC	F(null)	F	HWE
NIG	MHC-T	234-238	15	3	2.406	2.99	0.333	0.584	0.519	0.259	0.430	*
	MCW0312	210-218	19	3	1.667	2.42	0.474	0.400	0.339	-0.063	-0.183	ns
	MHC-D	307-319	20	5	3.265	4.26	0.750	0.694	0.646	-0.025	-0.081	*
	LEI0258	193-417	20	12	9.091	8.83	0.950	0.890	0.880	-0.036	-0.067	**
	MCW0371	200-210	19	6	2.407	4.40	0.368	0.584	0.547	0.270	0.370	ns
	MCW0370	174-180	18	7	3.560	5.72	0.167	0.719	0.691	0.618	0.768	***
IR	MHC-T	234-238	15	2	1.867	2.00	0.600	0.464	0.357	-0.127	-0.292	ns
	MCW0312	214-218	16	2	1.438	1.99	0.375	0.305	0.258	-0.102	-0.231	ns
	MHC-D	307-316	16	4	3.391	3.94	0.813	0.705	0.653	-0.081	-0.152	ns
	LEI0258	193-443	15	7	5.294	6.21	0.933	0.811	0.786	-0.081	-0.151	ns
	MCW0371	202-209	16	5	3.030	4.21	0.438	0.670	0.612	0.220	0.347	*
	MCW0370	172-182	16	7	3.710	5.47	0.625	0.730	0.692	0.082	0.144	ns
Ab	MHC-T	234-238	16	2	2.000	2.00	0.500	0.500	0.375	0.000	0.000	ns
	MCW0312	214-218	17	2	1.562	2.00	0.471	0.360	0.295	-0.133	-0.308	ns
	MHC-D	310-316	17	3	2.028	2.47	0.412	0.507	0.405	0.113	0.188	ns
	LEI0258	193-443	17	8	3.284	5.59	0.706	0.696	0.657	-0.002	-0.015	ns
	MCW0371	202-208	16	5	2.300	4.16	0.250	0.500	0.532	0.416	0.500	*
	MCW0370	175-182	17	4	2.639	3.41	0.471	0.621	0.544	0.113	0.242	**
Cobb	MHC-T	234-238	14	2	2.000	2.00	0.571	0.500	0.375	-0.067	-0.143	ns
	MCW0312	214-218	17	2	1.637	2.00	0.529	0.389	0.314	-0.152	-0.360	ns
	MHC-D	307-316	17	4	3.420	3.94	0.765	0.708	0.654	-0.047	-0.081	*
	LEI0258	193-385	17	9	5.898	7.12	0.706	0.830	0.810	0.087	0.150	ns
	MCW0371	202-209	17	5	3.503	4.73	0.412	0.715	0.674	0.270	0.424	**
	MCW0370	174-181	16	5	2.381	3.76	0.500	0.580	0.513	0.089	0.138	ns
Ross	MHC-T	234-238	16	2	1.932	2.00	0.563	0.482	0.366	-0.077	-0.166	ns
	MCW0312	214-218	15	2	1.220	1.91	0.200	0.180	0.164	-0.046	-0.111	ns
	MHC-D	307-316	15	4	2.381	3.76	0.733	0.580	0.535	-0.137	-0.264	ns
	LEI0258	193-461	16	8	3.737	6.37	0.938	0.732	0.710	-0.159	-0.280	ns
	MCW0371	202-209	16	5	2.462	3.89	0.313	0.594	0.658	0.389	0.474	*
	MCW0370	172-182	16	7	2.768	4.50	0.563	0.639	0.572	0.072	0.119	*
HL	MHC-T	234-238	18	2	1.314	1.96	0.278	0.239	0.211	-0.071	-0.161	ns
	MCW0312	214-218	19	2	1.232	1.90	0.211	0.188	0.171	-0.050	-0.118	ns
	MHC-D	307-316	19	3	1.306	2.32	0.263	0.234	0.216	-0.064	-0.124	ns
	LEI0258	307-479	19	6	2.854	4.48	0.632	0.650	0.606	0.026	0.028	ns
	MCW0371	205-208	18	3	2.031	2.44	0.389	0.508	0.404	0.132	0.234	ns
	MCW0370	174-179	19	5	3.390	4.36	0.263	0.705	0.661	0.470	0.627	***
LO	MHC-T	234-238	19	2	1.054	1.42	0.053	0.051	0.050	-0.006	-0.027	ns
	MCW0312	214-218	18	2	1.600	2.00	0.500	0.375	0.305	-0.142	-0.333	ns
	MHC-D	307-316	18	3	2.561	2.99	0.722	0.610	0.541	-0.069	-0.185	ns
	LEI0258	249-309	20	3	2.572	2.99	0.700	0.611	0.543	-0.048	-0.145	ns
	MCW0371	205-208	20	4	2.540	3.53	0.450	0.606	0.622	0.131	0.258	***
	MCW0370	174-178	20	4	3.556	3.88	0.150	0.719	0.665	0.651	0.791	***

[1] Populations abbreviations are defined in Table S2.

Table S5. Shared MS marker (Set 2) haplotypes in 29 populations.

Haplotype ID	allele order for six MS marker						Shared population [1] (relative haplotype frequency %)
	MHC_T	MCW0312	MHC-D	LEI0258	MCW0371	MCW0370	
MHC-B_MS1 (B2)	234	307	218	261	206	178	ADOL, TA (3.1%), Cobb (3.6%)
MHC-B_MS2 (B15)	234	310	218	261	203	NA	ADOL, KA (2.6%),
MHC-B_MS3 (B5)	234	313	218	295	209	NA	ADOL, WL (23.5%), NG (1.0%), Cobb (7.1%)
MHC-B_MS4 (B13)	234	316	218	205	202	179	ADOL, Ab (3.3%),
MHC-B_MS5 (B19)	236	316	218	539	205	176	ADOL, WL (5.9%)
MHC-B_MS7 (B21)	238	310	218	357	205	179	ADOL, RIR (17.9%), YCC (8.5%), CC1 (21.1%), CC2 (17.5%), CC3 (9.4%), CC4(5%), ND (2.9%), NN (21.4%), AS (3.3%), KA (2.6%), TA (1.6%), IR (10.7%), Ab (3.3%), Cobb (17.9%), Ross (3.3%)
MHC-B_MS8	232	307	218	309	205	174	RIR (7.1%), ND (2.9%)
MHC-B_MS9	238	316	218	295	208	179	RIR (21.4%), YCC (2.1%)
MHC-B_MS10	234	307	218	309	205	174	RIR (53.6%), NL (30.2%), NR (24.1), NW (3.3%), NY (5.6%), YCC (25.5%), CC1(23.7%), CC2 (15.2%), CC3 (18.8%), CC4 (12.5%), HI (8.8%), ND (1.4%), KA (7.9%), NU (5.9%), TA (15.6%), HL (11.4%), LO (8.8%)
MHC-B_MS22	234	310	214	249	206	178	NG (21.0%), NL (1.0%), NR (5.2%), NW (10.0%), NY (16.7%), NO (12.9%), YOG (2.6%), YCC (10.6%), LO (20.6%)
MHC-B_MS23	234	316	214	443	206	178	NG (10.0%), NY (4.2%), NO (1.6%), YOG (3.5%), NU (2.9%)
MHC-B_MS32	238	316	218	417	205	179	NG (1.0%), NR (1.7%), YCC (1.1%)
MHC-B_MS37	234	316	214	443	205	179	NG (4.0%), NR (10.3%), NW (1.7%), NY (5.6%), YOG (1.8%), YCC (3.2%), CC1 (7.9%), CC2 (22.5%), CC3 (6.3%), TA (1.6%)
MHC-B_MS38	234	313	214	193	202	178	NG (4.0%), NY (1.4%)
MHC-B_MS40	234	313	214	193	202	179	NG (1.0%), NR (5.2%), NY (6.9%), YCC (2.1%), Ab (6.7%), Ross (3.3%)
MHC-B_MS45	238	316	218	417	206	178	NG (4.0%), NIG (6.7%)
MHC-B_MS52	234	313	218	295	209	178	NG (2.0%), NY (2.8%)
MHC-B_MS57	238	316	218	417	206	179	NG (1.0%), NIG (3.3%)
MHC-B_MS59	234	307	218	309	206	178	NG (1.0%), LO (2.9%)
MHC-B_MS63	238	307	214	193	203	172	NL (9.4%), NR (5.2%), NW (3.3%), NY (20.8%), YCC (3.2%), CC4 (2.5%), NU (2.9%), IR (14.3%)
MHC-B_MS67	234	310	214	249	207	178	NL (1.0%), NW (1.7%), NY (2.8%), LO (2.9%)
MHC-B_MS71	234	310	214	249	206	177	NL (1.0%), NW (18.3%), NY (1.4%), YOG (0.9%), LO (2.9%)
MHC-B_MS72	234	307	218	309	205	172	NL (3.1%), NR (1.7%), NY (5.6%), YCC (1.1%), CC4 (2.5%)
MHC-B_MS76	234	307	218	309	206	174	NL (1.0%), NY (2.8%), YCC (8.5%), CC2 (2.5%), HI (2.9%), AS (3.3%), TA (1.6%), LO (5.9%)
MHC-B_MS77	238	307	214	193	202	172	NL (1.0%), NY (5.6%)
MHC-B_MS80	234	316	218	307	208	174	NL (1.0%), LO (8.8%)

[1] Populations abbreviations are defined in Table S2.

Table S5. Shared MS marker (Set 2) haplotypes in 29 population (continued).[1] Populations abbreviations are defined in Table S2.

Haplotype ID	allele order for six MS marker						Shared population [1] (relative haplotype frequency %)
	MHC_T	MCW0312	MHC-D	LEI0258	MCW0371	MCW0370	
MHC-B_MS82	234	310	218	193	203	181	NR (12.1%), NW (1.7%)
MHC-B_MS83	234	316	218	307	208	175	NR (6.9%), YOG (4.4%), CC4 (22.5%), KA (5.3%), NU (5.9%), TN (3.1%), NIG (6.7%), HL (30.6%), LO (26.5%)
MHC-B_MS84	234	316	218	309	208	175	NR (1.7%), CC4(2.5%), HL (5.6%)
MHC-B_MS86	234	316	218	307	208	178	NR (1.7%), LO (11.8%)
MHC-B_MS87	238	316	218	307	208	175	NR (1.7%), LO (2.9%)
MHC-B_MS95	238	316	214	478	205	177	NW (16.7%), NY (1.4%)
MHC-B_MS97	238	316	214	478	206	177	NW16.7%), NY (1.4%)
MHC-B_MS100	238	316	214	478	205	176	NW (3.3%), NY (2.8%)
MHC-B_MS101	234	316	218	249	202	NA	NY (2.8%), NO (14.5%), YOG (10.5)
MHC-B_MS103	234	316	214	443	205	178	NY (2.8%), YOG (0.9%), CC2(2.5%), CC3(6.3%), TA (1.6%)
MHC-B_MS107	234	316	218	249	202	178	NO (17.7%), YOG (7.0%)
MHC-B_MS108	234	313	218	417	206	178	NO (22.6%), YOG (2.6%)
MHC-B_MS109	236	313	218	417	206	178	NO (12.9%), YOG (17.5%)
MHC-B_MS110	236	313	218	417	206	177	NO (1.6%), YOG (0.9%)
MHC-B_MS111	236	307	218	247	203	177	NO (1.6%), YOG (2.6%)
MHC-B_MS112	234	316	218	249	202	177	NO (1.6%), YOG (3.5%)
MHC-B_MS131	238	310	214	217	204	NA	YOG (1.8%), ND (1.4%)
MHC-B_MS139	238	313	218	381	206	181	YCC (4.3%), CC2(2.5%), CC3(3.1%), KA (5.3%), IR (25.0%), Ab (30.0%), Ross (30.0%)
MHC-B_MS140	238	310	218	345	205	179	YCC (3.2%), TA (4.7%)
MHC-B_MS141	234	313	218	295	209	174	YCC (7.4%), CC4(2.5%)
MHC-B_MS142	234	307	218	307	205	174	YCC (1.1%), TA (1.6%)
MHC-B_MS143	234	313	218	295	209	181	YCC (1.1%), IR (3.6%), Ross (6.7%)
MHC-B_MS146	238	310	218	357	206	179	YCC (1.1%), CC1(2.6%), Ross (3.3%), HL (2.8%)
MHC-B_MS147	238	313	218	381	206	179	YCC (1.1%), Ross (3.3%)
MHC-B_MS148	234	313	218	295	209	179	YCC (3.2%), Cobb (7.1%), Ross (3.3%)
MHC-B_MS149	238	313	218	381	206	182	YCC (1.1%), IR (3.6%), AB (13.3%)
MHC-B_MS151	238	316	214	479	205	177	YCC (1.1%), HL (8.3%)
MHC-B_MS155	238	310	218	357	205	178	CC1(7.9%), CC3(3.1%)
MHC-B_MS156	234	316	210	273	201	178	CC1 (13.2%), CC2 (10.0%), CC3 (15.6%)
MHC-B_MS157	238	310	214	217	204	174	CC1(2.6%), CC2 (2.5%), CC3 (3.1%)
MHC-B_MS158	234	307	218	309	204	174	CC1 (2.6%), CC3 (3.1%)
MHC-B_MS159	238	310	214	217	204	179	CC1 (5.3%), CC2 (5.0%), CC3 (3.1%)
MHC-B_MS160	238	310	218	357	204	179	CC1 (2.6%), ND (1.4%), NN (6.0%), KA (2.6%)

Table S5. Shared MS marker (Set2) haplotypes in 29 populations (continued).

Haplotype ID	allele order for six MS marker						Shared population [1] (relative haplotype frequency %)
	MHC_T	MCW0312	MHC-D	LEI0258	MCW0371	MCW0370	
MHC-B_MS161	234	316	214	443	204	179	CC1 (2.6%), CC2 (6.3%), CC3 (6.3%)
MHC-B_MS171	238	310	218	217	204	179	CC3 (6.3%), ND (1.4%)
MHC-B_MS172	238	313	218	381	205	181	CC3 (3.1%), IR (3.6%), Ross (3.3%)
MHC-B_MS181	234	307	218	205	205	179	CC4 (2.5%), JF (3.3%), IR (3.6%), Cobb (3.6%), Ross (6.7%)
MHC-B_MS183	234	313	218	295	208	175	CC4 (5.0%), KA (2.6%), NIG (3.3%)
MHC-B_MS184	238	316	214	474	205	177	CC4 (5.0%), KA (2.6%)
MHC-B_MS185	234	316	218	443	205	179	CC4 (2.5%), IR (7.1%), Ab (6.7%), Ross (6.7%)
MHC-B_MS186	234	313	218	295	209	177	CC4 (2.5%), NIG (6.7%)
MHC-B_MS191	236	316	218	379	206	178	HI (1.5%), KA 2.6%)
MHC-B_MS199	234	307	214	249	202	178	HI (1.5%), ND (1.4%)
MHC-B_MS202	234	316	214	357	205	178	HI (2.9%), NIG (3.3%)
MHC-B_MS203	234	313	214	249	206	168	HI (1.5%), NN (1.2%)
MHC-B_MS205	234	316	214	357	206	178	HI (2.9%), NN (1.2%)
MHC-B_MS206	234	316	218	379	205	178	HI (2.9%), TA (1.6%)
MHC-B_MS210	236	310	214	249	205	168	HI (2.9%), ND (1.4%), NN (34.5%)
MHC-B_MS213	238	310	218	205	205	178	HI (4.4%), ND (2.9%)
MHC-B_MS216	238	307	214	249	202	168	HI (1.5%), ND (1.4%)
MHC-B_MS246	234	316	218	193	201	168	ND (1.4%), NN (3.6%)
MHC-B_MS248	234	316	218	193	201	179	ND (1.4%), NN (1.2%)
MHC-B_MS273	234	310	212	205	204	179	AS (3.3%), KA (2.6%)
MHC-B_MS275	234	307	218	307	206	178	AS (3.3%), TA (1.6 %)
MHC-B_MS291	234	307	218	261	205	179	KA (2.6%), TA (1.6%)
MHC-B_MS305	234	313	210	271	202	178	KA (2.6%), TA (1.6%)
MHC-B_MS326	234	310	218	249	206	178	NU (2.9%), TA (1.6%)
MHC-B_MS340	238	310	214	285	202	179	TA (1.6%), Cobb (10.7%)
MHC-B_MS363	234	316	218	307	208	177	TA (1.6%), HL (5.6%), LO (2.9%)
MHC-B_MS378	234	316	218	443	206	179	IR (7.1%), Ab (6.7%), Ross (3.3%)
MHC-B_MS379	234	307	218	205	206	176	IR (7.1%), Cobb (3.6%), Ross (3.3%)
MHC-B_MS388	234	316	218	321	208	175	Ab (3.3%), HL (2.8%)
MHC-B_MS389	234	316	214	369	206	179	Ab (6.7%), Cobb (3.6%), Ross (3.3%)

[1] Populations abbreviations are defined in Table S2.
